# Effectiveness, safety, and costs of thromboprophylaxis with enoxaparin or unfractionated heparin in inpatients with obesity

**DOI:** 10.3389/fcvm.2023.1163684

**Published:** 2023-06-16

**Authors:** Alpesh Amin, Alex Kartashov, Wilson Ngai, Kevin Steele, Ning Rosenthal

**Affiliations:** ^1^ Department of Medicine, University of California at Irvine, Irvine, CA, United States; ^2^ PINC AI™ Applied Sciences, Premier Inc., Charlotte, NC, United States; ^3^ Sanofi, Bridgewater, NJ, United States

**Keywords:** thromboprophylaxis, obesity, medical inpatients, enoxaparin, unfractionated heparin, cost analyses, bleeding, venous thromboembolism (VTE)

## Abstract

**Background:**

Obesity is a frequent and significant risk factor for venous thromboembolism (VTE) among hospitalized adults. Pharmacologic thromboprophylaxis can help prevent VTE, but real-world effectiveness, safety, and costs among inpatients with obesity are unknown.

**Objective:**

This study aims to compare clinical and economic outcomes among adult medical inpatients with obesity who received thromboprophylaxis with enoxaparin or unfractionated heparin (UFH).

**Methods:**

A retrospective cohort study was performed using the PINC AI™ Healthcare Database, which covers more than 850 hospitals in the United States. Patients included were ≥18 years old, had a primary or secondary discharge diagnosis of obesity [International Classification of Diseases (ICD)-9 diagnosis codes 278.01, 278.02, and 278.03; ICD-10 diagnosis codes E66.0*x*, E66.1, E66.2, E66.8, and E66.9], received ≥1 thromboprophylactic dose of enoxaparin (≤40 mg/day) or UFH (≤15,000 IU/day) during the index hospitalization, stayed ≥6 days in the hospital, and were discharged between 01 January 2010, and 30 September 2016. We excluded surgical patients, patients with pre-existing VTE, and those who received higher (treatment-level) doses or multiple types of anticoagulants. Multivariable regression models were constructed to compare enoxaparin with UFH based on the incidence of VTE, pulmonary embolism (PE)­­­­­­­­–related mortality, overall in-hospital mortality, major bleeding, treatment costs, and total hospitalization costs during the index hospitalization and the 90 days after index discharge (readmission period).

**Results:**

Among 67,193 inpatients who met the selection criteria, 44,367 (66%) and 22,826 (34%) received enoxaparin and UFH, respectively, during their index hospitalization. Demographic, visit-related, clinical, and hospital characteristics differed significantly between groups. Enoxaparin during index hospitalization was associated with 29%, 73%, 30%, and 39% decreases in the adjusted odds of VTE, PE-related mortality, in-hospital mortality, and major bleeding, respectively, compared with UFH (all *p *< 0.002). Compared with UFH, enoxaparin was associated with significantly lower total hospitalization costs during the index hospitalization and readmission periods.

**Conclusions:**

Among adult inpatients with obesity, primary thromboprophylaxis with enoxaparin compared with UFH was associated with significantly lower risks of in-hospital VTE, major bleeding, PE-related mortality, overall in-hospital mortality, and hospitalization costs.

## 1. Introduction

Venous thromboembolism (VTE), consisting of deep venous thrombosis (DVT) and pulmonary embolism (PE), is a leading cause of preventable hospital death and morbidity in the United States and worldwide (1, 2). In the United States, where more than 50% of VTE events occur among hospitalized patients, the estimated cost of treating a single VTE event is 2014US$12,000–$15,000, while managing VTE-associated complications costs approximately 2014US$18,000–$23,000 (3, 4). Primary pharmacologic thromboprophylaxis is well tolerated and cost-effective and significantly reduces VTE incidence among at-risk inpatients, according to the results of randomized clinical trials, prospective cohort studies, and systematic reviews and meta-analyses (5–10). Primary thromboprophylaxis is recommended for inpatients by professional organizations such as the American College of Chest Physicians, American Society of Hematology (ASH), American Heart Association, and International Society on Thrombosis and Haemostasis (ISTH) and by healthcare quality and accreditation groups such as the Agency for Healthcare Research and Quality, National Quality Forum, and the Joint Commission (1, 4, 11–14).

Inpatients usually receive thromboprophylaxis with either unfractionated heparin (UFH) or a low-molecular-weight heparin (LMWH) product. Compared with UFH, LMWH products such as enoxaparin are often more effective at reducing VTE risk and are associated with a lower risk of relevant side effects, such as major bleeding and heparin-induced thrombocytopenia (HIT) (15–20). In addition, LMWH products have a longer duration of the anticoagulant effect, which allows for once- or twice-daily dosing, and their predictable bioavailability and pharmacokinetics permit fixed dosing without the need for laboratory monitoring (21, 22). However, the longer duration of action of LMWH products and the fact that they are less easily inactivated by protamine sulfate make it more difficult to rapidly stop therapy (21). In contrast, UFH has a fast onset of action and undergoes rapid clearance from the circulation, allowing for more flexible titration of dosing and faster cessation of anticoagulation after treatment is stopped. Administering protamine sulfate also rapidly reverses UFH activity (23). Because UFH does not undergo substantial renal clearance, it can be used in patients with kidney failure or chronic kidney disease (24). However, the short half-life of UFH means that continuous infusion is usually necessary to achieve therapeutic levels of anticoagulation (23). In addition, because UFH has a highly variable dose–response relationship, patients need to be monitored by measuring activated partial thromboplastin time, anti-factor Xa activity, or activated clotting time (25–27).

Obesity [body mass index (BMI) ≥ 30 kg/m^2^] is a significant risk factor for VTE (28–33) that has been incorporated into VTE risk assessment tools (10, 34). Obesity appears to increase VTE risk through several mechanisms, including sedentariness, increased intra-abdominal pressure, decreased blood velocity in the lower limbs, and inflammatory and metabolic abnormalities that lead to a hypercoagulable state (35–37). Most observational studies of individuals both with and without obesity have shown that higher BMI is positively associated with VTE incidence (29, 32, 38–40). Obesity also is a significant risk factor for hospital-associated VTE, including among medically ill inpatients (33, 41), but few studies have compared the safety, effectiveness, and costs of different thromboprophylactic agents among inpatients with obesity. To garner insights on best practices, we compared real-world clinical and economic outcomes among adult medically ill inpatients in the United States who had a primary or secondary diagnosis of obesity and received thromboprophylaxis with enoxaparin or UFH during their hospital stay.

## 2. Methods

### 2.1. Study design and data source

This was a retrospective cohort study of the PINC AI™ Healthcare Database (PHD), a service-level, all-payer database of more than 850 urban and rural non-profit, non-governmental, community and teaching hospitals, and health systems in 45 states and the District of Columbia that covers approximately 25% of annual admissions in the United States (42). Each patient in the PHD is assigned a unique masked identifier to track encounters within the same hospital system. Data are extracted from standard hospital discharge files and include demographic information, admission and discharge diagnoses, comorbidities, and date-stamped, billed items linked to medications, medical services, procedures, laboratory tests, microbiology tests, diagnostic and therapeutic services, and disposition and discharge health status. Medication data are available for each day of the hospital stay and include medication type, dose, cost per dose, and quantity.

### 2.2. Patients

The study population consisted of adults in the United States aged 18 years or older with an inpatient medical admission between January 1, 2010, and September 30, 2016, a primary or secondary discharge code for obesity [International Classification of Diseases (ICD), Ninth Revision, Clinical Modification (CM) codes: 278.01, 278.02, or 278.03; or ICD, Tenth Revision, CM codes: E66.0*x*, E66.1, E66.2, E66.8, or E66.9], and a hospital stay of at least 6 days (the index hospitalization period), during which at least one thromboprophylactic dose of UFH (≤15,000 IU/day) or enoxaparin (≤40 mg/day) was administered as per the hospital's chargemaster (a central repository of charges and associated coding data). Patients who were readmitted to the same hospital system for any reason within 90 days after discharge (readmission period) were also identified and evaluated. All data were de-identified, Health Insurance Portability and Accountability Act (HIPAA) compliant, and considered to be exempt from institutional review board oversight as per 45 CFR 46.101(b) (4).

Surgical patients were excluded from this study because they are an inherently different patient population. To reduce the likelihood of confounding or bias, patients also were excluded if they met certain criteria during the index hospitalization period or the 90 days prior to admission (pre-index hospitalization period). Key exclusion criteria were receipt of enoxaparin, UFH, fondaparinux, dalteparin, or rivaroxaban during the pre-index hospitalization period; a diagnosis of VTE during the pre-index hospitalization period or the first 2 days of the index hospital admission; and receipt of therapeutic-dose anticoagulants during the first 2 days of hospital admission. Additional exclusionary criteria were receipt of warfarin, dabigatran, apixaban or edoxaban, mechanical VTE prophylaxis, surgery, or obstetric procedures; pregnancy; a diagnosis of a thrombophilic condition, a hemorrhagic disorder, or an active peptic ulcer; or the administration of any combination of enoxaparin and UFH [with or without another anticoagulant (e.g., fondaparinux, dalteparin, or rivaroxaban)] during the pre-index or index hospitalization periods. Patients with missing cost data also were excluded from the study.

### 2.3. Outcome measures

The principal clinical outcome measure was a VTE event during the index hospitalization or 90-day readmission periods, with VTE defined as a primary or secondary discharge ICD-9/10-CM diagnosis code for DVT (ICD-9-CM: 451.xx–453.xx; ICD-10-CM: I80.xxx–I82.xxx) or PE (ICD-9-CM: 415.1x; ICD-10-CM: I26.9x and T80.xxxx–T82.xxxx). Secondary clinical outcome measures were in-hospital mortality, PE-related mortality, major bleeding, and HIT during the index hospitalization and readmission periods. Major bleeding was defined as a primary or secondary discharge ICD-9/10-CM diagnosis code for serious bleeding (ICD-9-CM: 430, 431, 432.x, 459.0, 578.x, 786.3x; ICD-10-CM: I60.9–I62.9, R58, K92.0, K92.2, R04.x, D75.82). Heparin-induced thrombocytopenia was defined as a primary or secondary discharge ICD-9/10-CM diagnosis code for HIT (ICD-9-CM: 289.84; ICD-10-CM: D75.82).

Economic outcome measures consisted of total hospitalization costs for the index hospitalization and readmission periods and the cost of pharmacologic prophylaxis (the combined cost of all doses of enoxaparin or UFH) during the index hospitalization period, as determined based on hospital chargemaster data. All cost calculations were adjusted to 2017 US dollars based on consumer price index for all urban consumers for hospitals and related services.

Covariates were assessed to identify possible confounders of the relationship between thromboprophylaxis and study outcomes. These covariates included patient demographics (age, sex, race, and payor type), visit characteristics [admission type and source, discharge disposition, intensive care unit (ICU) admissions], and relevant comorbidities. The Charlson–Deyo comorbidity index (CCI) was assessed by using the Premier-modified Charlson–Deyo algorithm of primary and secondary ICD-10 diagnosis and procedure codes at discharge for the index visit (43). The CCI assessment included myocardial infarction, congestive heart failure, peripheral vascular disease, cerebrovascular disease, chronic pulmonary disease, rheumatic disease, diabetes, moderate or severe renal disease, malignancies, liver diseases, metastatic solid tumors, and HIV disease. The mean and median of the CCI score were reported; ICD-10 codes for each condition are listed in the Supplementary Material. In addition to the CCI comorbidities, congestive heart failure, myocardial infarction, chronic obstructive pulmonary disease (COPD), lower limb fracture, inflammatory bowel disease, intubation, malignant hypertension, and nephrotic syndrome were also assessed individually. These comorbidities were selected as covariates because they are known risk factors for the outcomes of interest and may be associated with the exposure being evaluated (enoxaparin versus UFH). The 3M™ All Patient Refined™ Diagnosis-Related Group (APR™-DRG) Severity of Illness (APR-DRG-SOI) score was also assessed. This score is categorized as minor, moderate, major, or extreme and incorporates patient age, procedures, and the clinical severity of the primary diagnosis and all secondary diagnoses at the time of hospital discharge (44). Hospital characteristics were evaluated, including bed size (number), geographic region, population served (rural or urban), and teaching status (teaching or non-teaching).

### 2.4. Statistical analyses

Analyses were performed with SAS v. 9.4 (SAS Institute Inc., Cary, NC, USA). Patients were grouped according to whether they had received thromboprophylactic enoxaparin or UFH during the index hospitalization period. Descriptive statistics were reported as mean ± standard deviation (SD) for continuous variables and proportions and frequencies for categorical variables. Bivariate analyses were performed to compare demographics and visit, clinical, and hospital characteristics between the enoxaparin and UFH groups. Student's *t*-test and the Wilcoxon rank sum test were used for comparisons of continuous variables, and the *χ*^2^ test was used for comparisons of categorical variables between the enoxaparin and UFH groups. To determine which tests to apply for statistical significance, normality of data was evaluated by histogram and by the Kolmogorov–Smirnov test.

Multivariable logistic regression models were constructed to determine the estimated odds of VTE, in-hospital mortality, PE-related mortality, and major bleeding between the enoxaparin and UFH groups for both the index hospitalization period and the 90-day readmission period. These models were adjusted for confounders, including patient demographics, visit and hospital characteristics, CCI categories, severity indicators (APR-DRG-SOI, ICU stay), and clinically relevant comorbidities (myocardial infarction, inflammatory bowel disease, nephrotic syndrome, fracture of lower limb, COPD, intubation, and malignant hypertension). Creatinine levels were not included in the study dataset and therefore were not adjusted for.

Unadjusted means ± SDs were calculated for total hospitalization costs per patient, pharmacologic prophylaxis costs per patient during the index hospitalization period, and total hospitalization costs per patient during the 90-day readmission period. To minimize the impact of outliers, costs were winsorized at the 2.5th percentile and the 99th percentile (those that were less than the 2.5th percentile were assigned the value of the 2.5th percentile, and those that were greater than the 99th percentile were assigned the value of the 99th percentile). Generalized linear models with gamma link function were created to estimate the adjusted costs for each group, and the results were presented as adjusted means and confidence intervals (CIs). All regression models were evaluated for the fitness and convergence of algorithms. Regression diagnostics (performed to evaluate multicollinearity between variables) indicated that there was no need to delete any variable. For all analyses for which *p*-values were calculated, statistical significance was defined as *p* < 0.05.

## 3. Results

A total of 117,630 hospitalized adults in the United States with obesity were identified, of whom 67,193 (57%) met the selection criteria. In all, 44,367 (66%) of these patients received thromboprophylactic enoxaparin, and 22,826 (33%) received thromboprophylactic UFH. Table 1 compares the demographic, clinical, visit, and hospital characteristics between the two groups. Relatively small but statistically significant between-group differences were found for all variables except the proportion of patients with a diagnosis of diabetes and the proportion of patients with hemiplegia or paraplegia. The mean age was 2 years younger in the enoxaparin group than in the UFH group (59 ± 14 years vs. 61 ± 14 years, respectively). Patients in the enoxaparin group were more likely to be female (61% vs. 55% in the UFH group), White (72% vs. 67%, respectively), admitted from home (81% vs. 77%), discharged to home (77% vs. 71%), and admitted to non-teaching hospitals (65% vs. 46%) of 1–299 beds (38% vs. 32%) in rural areas (13% vs. 9%) of the Southern United States (56% vs. 36%) (all *p* < 0.0001). Patients in the enoxaparin group also were less likely to be Medicare beneficiaries (51% vs. 55% in the UFH group), to have an extreme APR-DRG-SOI score (17% vs. 24%, respectively), to have a CCI score ≥3 (39% vs. 52%), and to be admitted to the ICU during their index hospitalization period (27% vs. 35%) (all *p* < 0.0001). Mean hospital lengths of stay were 8.6 days in the enoxaparin group and 9 days in the UFH group (*p* < 0.0001).

**Table 1:** Demographic, clinical, and hospital characteristics of adult inpatients with obesity in the United States who received thromboprophylaxis with enoxaparin or unfractionated heparin.

Characteristics (%)	Enoxaparin (*N* = 44,367)	Unfractionated heparin (*N* = 22,826)	*p*-Value
Demographic characteristics			
Age (years)	59 ± 14	61 ± 14	<0.0001
Female sex	26,961 (61)	12,474 (55)	<0.0001
Race			<0.0001
White	31,895 (72)	15,305 (67)	
Black	6,856 (15)	3,836 (17)	
Other	5,461 (12)	3,615 (16)	
Unknown	155 (0.33)	70 (0.31)	
Payor type			<0.0001
Private	10,946 (25)	5,570 (24)	
Medicaid	6,587 (15)	3,142 (14)	
Medicare	22,714 (51)	12,637 (55)	
Uninsured	3,377 (8)	1,228 (5)	
Unknown	743 (2)	249 (1)	
Visit characteristics			
Admission source			<0.0001
Home	17,662 (77)	35, 993 (81)	
Transfer from acute care facility	2,671 (12)	3,695 (8)	
Transfer from skilled nursing facility	451 (2)	715 (2)	
Emergency room	1,117 (5)	2,316 (5)	
Other/unknown	925 (4)	1,648 (4)	
Admission type			<0.0001
Emergency	33,679 (76)	16,480 (72)	
Urgent	6,784 (15)	3,760 (16)	
Elective	3,572 (8)	2,345 (10)	
Trauma	99 (0.2)	134 (0.2)	
Unknown	233 (0.5)	107 (0.5)	
Discharge status			<0.0001
Expired	999 (4)	1,037 (2.3)	
Home	16,236 (71)	34,033 (77)	
Transferred to another acute care setting	457 (2)	736 (1.7)	
Transferred to nursing or rehabilitation facility	4,797 (21)	7,996 (18)	
Other	337 (1.5)	565 (1.3)	
ICU admission stay	12,158 (27)	8,005 (35)	<0.0001
Hospital length of stay (days)	8.6 ± 4.3	9 ± 4.9	<0.0001
Clinical characteristics			
Severity of illness			<0.0001
Minor	1,387 (3.1)	597 (2.6)	
Major	13,135 (30)	5,035 (22)	
Moderate	22,276 (50)	11,664 (51)	
Extreme	7,569 (17)	5,530 (24)	
CCI score^a^			<0.0001
0	4,630 (10)	1,777 (7.8)	
1–2	22,361 (50)	9,255 (41)	
≥3	17,376 (39)	11,794 (52)	
Myocardial infarction	3,513 (7.9)	2,618 (11)	<0.0001
Congestive heart failure	14,466 (33)	9,630 (42)	<0.0001
Peripheral vascular disease	2,452 (5.5)	1,732 (8)	<0.0001
Cerebrovascular disease	3,343 (7.5)	2,474 (11)	<0.0001
Dementia	1,384 (3.1)	793 (3)	0.0139
COPD	24,213 (55)	10,639 (47)	<0.0001
Rheumatologic disease			0.0002
Peptic ulcer disease	258 (0.6)	169 (0.7)	0.0141
Mild liver disease	417 (0.9)	271 (1.2)	0.0026
Diabetes	9,730 (43)	18,880 (43)	0.8568
Diabetes with chronic complications	5,155 (12)	3,486 (15)	<0.0001
Hemiplegia or paraplegia	1,494 (2.6)	642 (2.8)	0.0560
Renal disease	6,456 (15)	6,621 (29)	<0.0001
Any malignancy, including leukemia and lymphoma	2,844 (6.4)	1,716 (7.5)	<0.0001
Moderate or severe liver disease	156 (0.4)	133 (0.6)	<0.0001
Metastatic solid tumor	1,184 (2.7)	676 (3.0)	0.0284
AIDS/HIV	63 (0.3)	60 (0.1)	0.0005
Inflammatory bowel disease	266 (0.6)	109 (0.5)	0.0443
Fracture of lower limb	135 (0.3)	46 (0.2)	0.0149
Nephrotic syndrome	66 (0.2)	71 (0.31)	<0.0001
Intubation	4,444 (10)	3,096 (14)	<0.0001
Malignant hypertension	5,836 (13)	2,783 (12)	0.0004
HIV infection	160 (0.4)	115 (0.5)	0.0059
Hospital characteristics			
Geographic region			<0.0001
Northeast	5,442 (12)	6,776 (30)	
Midwest	8,071 (18)	4,725 (21)	
South	24,654 (56)	8,201 (36)	
West	6,200 (14)	3,124 (14)	
Bed size			<0.0001
1–299	16,739 (38)	7,363 (32)	
300–499	14,335 (32)	7,800 (34)	
≥500	13,293 (30)	7,663 (34)	
Population served			<0.0001
Rural	5,791 (13)	2,015 (9)	
Urban	38,576 (87)	20,811 (91)	
Teaching status			<0.0001
Non-teaching	28,738 (65)	10,483 (46)	
Teaching	12,343 (54)	15,629 (35)	

Data are presented as mean ± SD or *n* (%) unless otherwise indicated. AIDS, acquired immunodeficiency syndrome; CCI, Charlson Comorbidity Index; COPD, chronic obstructive pulmonary disease; HIV, human immunodeficiency virus; ICU, intensive care unit.

^a^ Myocardial infarction, congestive heart failure, peripheral vascular disease, history of cerebrovascular accident and transient ischemic attacks, dementia, chronic obstructive pulmonary disease, connective tissue disease, mild or moderate to severe liver disease, diabetes mellitus uncomplicated or with end-organ damage, hemiplegia, mild or moderate to severe renal disease, malignancy, and HIV-positive status.

Table 2 compares the clinical outcomes between the enoxaparin and UFH groups. For the index hospitalization period, unadjusted risks of VTE, overall in-hospital mortality, and PE-related mortality were 0.48%, 2.34%, and 0.02% in the enoxaparin group and 0.85%, 4.38%, and 0.11% in the UFH group (all *p* < 0.0001), respectively. In the multivariable analysis, thromboprophylaxis with enoxaparin vs. UFH was associated with significantly lower adjusted odds of a VTE event [adjusted odds ratio (aOR) 0.71, 95% confidence interval (CI) 0.57–0.88], in-hospital mortality (aOR 0.70, 95% CI 0.63–0.78), and PE-related mortality (aOR 0.27, 95% CI 0.17–0.61).

**Table 2:** Clinical outcomes of adult inpatients with obesity in the United States who received thromboprophylaxis with enoxaparin or unfractionated heparin during their index hospitalization.

	Enoxaparin	Unfractionated heparin	Enoxaparin (vs. unfractionated heparin)	
			Adjusted OR^a^ (95% CI)	*p*-Value
Index hospitalization period	*N* = 44,367	*N* = 22,826		
VTE event	212 (0.48)	195 (0.85)	0.71 (0.57–0.88)	0.0017
In-hospital mortality	1,037 (2.34)	999 (4.38)	0.70 (0.63–0.78)	<0.0001
PE-related mortality	9 (0.02)	25 (0.11)	0.27 (0.17–0.61)	0.0019
Major bleeding	764 (1.72)	798 (3.5)	0.61 (0.55–0.69)	<0.0001
90-day readmission period	*n* = 20,303 (46%)	*n* = 10,363 (45%)		
VTE event	542 (2.67)	328 (3.17)	0.88 (0.75–1.02)	0.0821
In-hospital mortality	561 (2.76)	329 (3.17)	1.07 (0.92–1.24)	0.4115
PE-related mortality	19 (0.09)	19 (0.18)	0.66 (0.33–1.32)	0.2407
Major bleeding	473 (2.33)	314 (3.03)	0.85 (0.73–1.00)	0.0521

Data are presented as *n* (%) unless otherwise indicated; CI, confidence interval; OR, odds ratio; PE, pulmonary embolism; VTE, venous thromboembolism.

^a^ Adjusted for patient characteristics (age, sex, race, payer); visit characteristics (admission source and type and ICU admission); clinical characteristics (severity of illness, Charlson Comorbidity Index score, myocardial infarction, inflammatory bowel disease, nephrotic syndrome, fracture of lower limb, chronic obstructive pulmonary disease, intubation, malignant hypertension); and hospital characteristics (teaching status, bed number category, geographic region, and rurality).

A total of 20,303 (46%) patients in the enoxaparin group and 10,363 (45%) patients in the UFH group were readmitted to the same hospital system within 90 days after index discharge. Among readmitted patients, risks of VTE, in-hospital mortality, and PE-related mortality were 2.67%, 2.76%, and 0.09% among those who had received enoxaparin and 3.17%, 3.17%, and 0.18% among those who had received UFH during their index hospitalization, respectively. None of these differences reached statistical significance in the multivariable analysis.

Major bleeding during the index hospitalization period was coded for 764 (1.7%) patients in the enoxaparin group and 798 (3.5%) patients in the UFH group; HIT was coded in 19 (0.04%) and 32 (0.14%) patients (both *p* < 0.0001) (Table 2). During the 90-day readmission period, major bleeding was coded in 473 (2.3%) patients who had received enoxaparin and 314 (3.0%) patients who had received UFH during their index hospitalization (*p* = 0.0002), while HIT was coded in 13 (0.06%) and 17 (0.16%) patients, respectively (*p* = 0.008). In multivariable analyses, thromboprophylaxis with enoxaparin was associated with significantly lower odds of major bleeding during index hospitalization (aOR 0.61, 95% CI 0.55–0.69, *p* < 0.0001).

Table 3 presents the unadjusted and adjusted mean total costs of hospitalization and pharmacologic prophylaxis during the index hospitalization period and the unadjusted and adjusted mean total cost of hospitalization during the 90-day readmission period. The adjusted mean cost of pharmacologic thromboprophylaxis during the index hospitalization period was 2017US$80 higher in the enoxaparin group than in the UFH group (2017US$147 [95% CI $137–$158] vs. 2017US$67 [95% CI $62–$72], *p* < 0.0001). However, the adjusted mean total cost of hospitalization per patient was significantly lower in the enoxaparin group than in the UFH group (2017US$17,374 [95% CI $16,769–$18,001] vs. $18,724 [95% CI $18,072–$19,400], *p* < 0.0001). Additionally, during the readmission period, patients who had received enoxaparin during their index hospitalization had a significantly lower adjusted mean total cost of hospitalization compared with patients who had received UFH (2017US$6,511 [95% CI 5,467–7,754] vs. 2017US$6,866 [95% CI 5,765–8,177], *p* = 0.007).

**Table 3:** Economic outcomes among adult inpatients with obesity in the United States who received thromboprophylaxis with enoxaparin or unfractionated heparin during their index hospitalization.

	Unadjusted mean costs			Adjusted mean estimates^a^		
	Enoxaparin	Unfractionated heparin	*p*-Value	Enoxaparin	Unfractionated heparin	*p*-Value
Index hospitalization period						
Total hospitalization cost	16,057± 13,040	20,330 ± 17,893	<0.0001	17,374 (16,769–18,001)	18,724 (18,072–19,400)	<0.0001
Cost of pharmacologic prophylaxis	192 ± 219	94 ± 132	<0.0001	147 (137–158)	67 (62–72)	<0.0001
90-day readmission period						
Total hospitalization cost	9,498 ± 24,871	11,420 ± 21,546	<0.0001	6,511 (5,467–7,754)	6,866 (5,765–8,177)	0.007

Costs are per patient. Data are presented in 2017US$ as mean ± standard deviation or mean (95% confidence interval) unless otherwise indicated.

^a^ Adjusted for patient characteristics (age, sex, race, payer); visit characteristics (admission source and type and ICU admission); clinical characteristics (severity of illness, Charlson comorbidity index score, myocardial infarction, inflammatory bowel disease, nephrotic syndrome, fracture of lower limb, chronic obstructive pulmonary disease, intubation, malignant hypertension); and hospital characteristics (teaching status, bed number category, geographic region, and rurality).

## 4. Discussion

To our knowledge, this is the first large administrative hospital database study to compare real-world outcomes and costs among adult inpatients with obesity in the United States who received thromboprophylaxis with enoxaparin or UFH. After adjusting for relevant covariates, the enoxaparin group had 29% lower odds of VTE, 30% lower odds of in-hospital death from any cause, 73% lower odds of PE-related mortality, and 39% lower odds of major bleeding compared with the UFH group during the index hospitalization period. Thromboprophylaxis with enoxaparin cost more than UFH, but the enoxaparin group had significantly lower adjusted mean total costs of hospitalization per patient, both during the index hospitalization period and the 90-day readmission period.

In this observational study of medical inpatients with obesity, thromboprophylaxis with enoxaparin versus UFH was associated with similar reductions in VTE risk and mortality as in randomized controlled trials of patients hospitalized with acute ischemic stroke, heart failure, and severe respiratory disease (16, 18, 19). The difference in outcomes between enoxaparin and UFH might be related to a greater antithrombotic effect of enoxaparin and its longer duration of the anticoagulant effect. Interestingly, the differences in outcomes between the enoxaparin and UFH groups in our study were even more pronounced than in a similarly designed study of general medically ill inpatients from the PHD (45). That study controlled for obesity, while both studies controlled for other comorbidities that are known VTE risk factors (see Supplementary Material). Therefore, the even stronger associations observed in the current study might reflect an even greater benefit from thromboprophylaxis with enoxaparin among patients with obesity compared with other medically ill inpatients.

**Figure 1:**
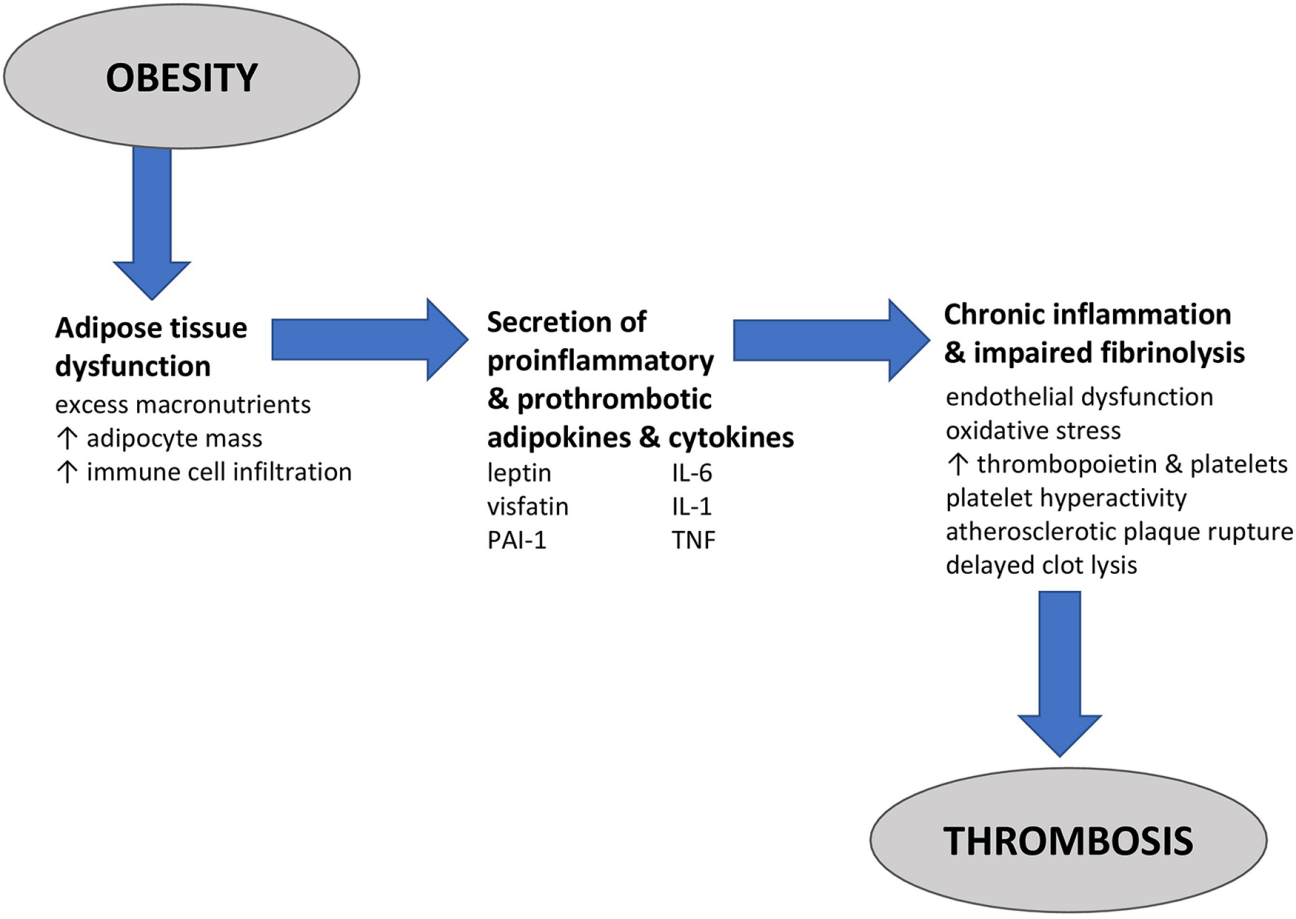
Pathophysiology of thrombosis in obesity. Obesity is characterized by adipose tissue dysfunction or changes in the adipose tissue microenvironment that promote pathology, including hypercoagulability. In adipose tissue dysfunction, an excess of macronutrients in the tissue microenvironment leads to greater adipocyte mass and the infiltration of immune cells, such as macrophages and T cells. These cells release pro-inflammatory, prothrombotic adipokines [leptin, visfatin, and plasminogen activator inhibitor 1 (PAI-1)] and cytokines [interleukin (IL)-6, IL-1, and tumor necrosis factor (TNF)]. The end result is a state of chronic systemic inflammation and impaired fibrinolysis in which oxidative stress, increased production of thrombopoietin and platelets, platelet hyperactivity, endothelial cell dysfunction, atherosclerotic plaque rupture, and delayed clot lysis increase the risk for thrombosis and venous thromboembolism.

Obesity is characterized by various metabolic and inflammatory changes that promote a hypercoagulable state (35–37) (Figure 1). Individuals with obesity are at risk for adipose tissue dysfunction, which can be defined as an excess of macronutrients in the tissue microenvironment that leads to greater adipocyte mass and the infiltration of immune cells, such as macrophages and T cells. This results in greater secretion of pro-inflammatory, prothrombotic adipokines [leptin, visfatin, plasminogen activator inhibitor 1 (PAI-1)] and cytokines [interleukin (IL)-6, IL-1, tumor necrosis factor], which promotes chronic inflammation and impairs fibrinolysis (46–49). Together, endothelial cell dysfunction, oxidative stress, increased production of thrombopoietin and platelets, platelet hyperactivity, atherosclerotic plaque rupture, and delayed clot lysis promote thrombosis. In one study of 109 consecutive patients with obesity (mean BMI 46.6 ± 7 kg/m^2^), 18% were classified as hypercoagulable based on rotational thrombelastometry, and these patients had increased levels of C-reactive protein, fibrinogen, and platelets compared with the other patients (37). Other studies also have found higher platelet counts among individuals with obesity compared with non-obese individuals, although the association sometimes was limited to women (50). Interestingly, weight loss after bariatric surgery is associated with reductions in platelet counts (51). In animal models, obesity has been linked to the overproduction of adipokines, such as leptin, that contribute to thrombosis (52). Genetic studies also back the association: In a large genome-wide association study of 68 variants associated with obesity, a genetic profile consistent with elevated BMI was associated with a 57% increase in VTE risk (OR 1.57, 95% CI 1.08–1.97) (53). A very similar odds ratio (1.59, 95% CI 1.30–1.93) was identified in a Mendelian randomization study of the relationship between genetically predicted BMI and risk for VTE (54).

Obesity is a pandemic. Worldwide, its prevalence has doubled since 1980, and in the United States, it is predicted that 50% of adults will have obesity and nearly 25% will have severe obesity (BMI ≥35) by 2030 (55–57). Despite the global prevalence of obesity and its robustness as a VTE risk factor, few studies have compared outcomes among inpatients with obesity who received thromboprophylaxis with different pharmacologic agents. Among those published, most were single-center experiences, clinical trials of surgery patients that excluded medical inpatients, or studies of dosing regimens for a single drug (58–64). Partly for this reason, clinical guidelines tend to note that obesity is a risk factor for VTE without offering specific recommendations for selecting thromboprophylactic agents and determining dosing of such agents in this setting (11, 65). An exception is a guidance document published in 2021 by the ISTH Scientific Subcommittee on Anticoagulation that addresses the use of direct-acting oral anticoagulants (DOACs) in patients with obesity; however, the authors note a relative dearth of data, especially on DOACs in patients with BMI > 40 kg/m^2^ (66). Gaps in knowledge and guidance could contribute to the underuse of antithrombotic agents for thromboprophylaxis in patients with obesity. Strikingly, in our dataset, 35% (41,169) of patients had no record of receiving any form of anticoagulation during their index hospitalization, even though every index hospitalization period lasted at least 6 days. Although our study was not specifically designed to assess gaps in care, these results suggest that the well-documented underuse of thromboprophylaxis in high-risk hospitalized medical patients (67–70) extends to inpatients with obesity.

In both the current study and the previously published PINC AI™ study of medically ill inpatients (45), thromboprophylaxis with enoxaparin versus UFH was associated with significantly lower adjusted odds of major bleeding. These findings are in line with those from a retrospective study of patients with obesity and BMI > 40 kg/m^2^ in which the risk of major bleeding was significantly higher with high-dose UFH thromboprophylaxis (7,500 units every 8 h) versus high-dose enoxaparin thromboprophylaxis (40 mg every 12 h) (63). To our knowledge, the current study and the study of high-dose enoxaparin thromboprophylaxis are among the only published comparisons of the safety of different thromboprophylactic agents among inpatients with obesity.

To our knowledge, this also is the first study of inpatients with obesity to compare economic outcomes with different thromboprophylactic agents. In our study, thromboprophylaxis with enoxaparin cost more than UFH but was associated with significantly lower total hospitalization costs. Specifically, patients in the enoxaparin group had a 2017US$1,351 lower adjusted mean total hospital cost per discharge during the index hospitalization period and a 2017US$355 lower adjusted mean total hospital cost per discharge during the readmission period. Other studies also have identified lower total hospitalization costs among high-risk inpatients who received thromboprophylaxis with enoxaparin as compared with UFH (71–76). Based on these findings, thromboprophylaxis with enoxaparin might help reduce the cost of managing acute VTE, which was estimated to be US$12,000–US$15,000 per patient in 2014 (3). However, we should be cautious about directly inferring an economic benefit from our findings. Although the multivariable regression analysis adjusted for differences in patient demographics and clinical characteristics, such as renal disease, between the two comparison groups, unmeasured or residual confounding may exist.

### 4.1. Limitations

Several limitations of our study warrant mention, many of which are inherent to retrospective studies of hospital administrative databases. First, the study population may not represent all obese inpatients in the United States, and potential selection bias may exist because the definition of obesity was based solely on ICD diagnosis codes, which may be underreported in administrative data (note that the PHD does not have BMI values) (77, 78). Second, the PHD lacks data on some risk factors for VTE (such as immobility and smoking status) and also lacks data on creatinine values, which makes it harder to determine whether the UFH group had a higher prevalence of acute renal failure [which may increase the risk for VTE (79) and increases elimination half-life and bleeding risk with LMWH (80)]. Therefore, unmeasured confounding could exist. Third, inaccurate or incomplete coding or the application of relatively unselected codes could have affected determinations of study eligibility, covariates, and outcomes. We note that risks of VTE, bleeding, and HIT were lower in our study than in some other clinical trials and observational studies (16, 17, 19, 20, 58), which could be due to underreporting or could reflect a true difference in the study populations. Lastly, because the PHD only tracks patient readmissions to the same hospital system, patients who were readmitted to another hospital system would have been lost to follow-up. Although this could have led to an underestimation of outcomes during follow-up, such underestimation is assumed to be non-differential between the comparison groups.

We also note that by design, our study would have excluded patients who received a weight-based dose or a higher fixed dose of enoxaparin, since either approach would have led to a daily dose exceeding 40 mg, the cutoff for inclusion in the enoxaparin group. Some studies have indicated that standard dosing with LMWHs, UFH, and DOACs may not achieve optimal thromboprophylaxis in patients with obesity, particularly patients with severe obesity, and that dose adjustment may therefore be warranted (58, 81–84). In small prospective studies, medical chart reviews, and pooled analyses, weight-based pharmacologic thromboprophylaxis was safe and helped achieve desired serum levels of anti-factor Xa activity in patients with obesity (61, 64, 85, 86). In other studies, the use of higher fixed doses outperformed standard-dose thromboprophylaxis (58, 84, 87). However, there is a lack of robust clinical and pharmacokinetic/pharmacodynamic data on these approaches, and experts from ASH, ISTH, and the National Institute for Health and Care Excellence have called for more research on the use of weight-based versus fixed dosing in patients with obesity (65, 66, 88). Because the PHD lacks data on BMI, we were unable to examine outcomes in a subgroup of patients with severe obesity, who might be at the highest risk for VTE and might be most likely to benefit from dose adjustment (32).

## 5. Conclusions

This first-in-kind large administrative hospital study offers a unique, real-world comparison of clinical and economic outcomes with thromboprophylactic enoxaparin or UFH among medical inpatients with obesity—a high-risk, understudied population. Compared with UFH, enoxaparin was associated with significantly lower odds of a VTE event, in-hospital mortality, PE-related mortality, and major bleeding during the index hospitalization period. Thromboprophylaxis with enoxaparin cost more than UFH but was associated with significantly lower total hospitalization costs both during the index hospitalization period and the 90-day readmission period. These findings may help guide clinicians and hospitals as they seek to optimize VTE prophylaxis for inpatients with obesity, a rapidly growing population worldwide.

## Data Availability

The data analyzed in this study is subject to the following licenses/restrictions: The PINC AI™ Healthcare Database is a proprietary HIPAA-compliant de-identified database. Requests to access the de-identified datasets must first be approved. Requests to access these datasets should be directed to NR, ning_rosenthal@premierinc.com.
