# Supplementary material for: Effectiveness, safety, and costs of thromboprophylaxis with enoxaparin or unfractionated heparin in inpatients with obesity
Source: Front Cardiovasc Med. 2023 Jun 16;10:1163684. doi: 10.3389/fcvm.2023.1163684 (PMC10313352; doi:10.3389/fcvm.2023.1163684)
Supplement: Supplementary file 1 [file Table1.docx]

Supplementary Material

# 1 Controlling for Comorbidities in Multivariable Analyses

Comorbidities were identified by the presence of a primary or secondary admission or discharge *International Classification of Diseases, Ninth Revision* (ICD)-9 or *Tenth Revision, Clinical Modification* (-10-CM) code during the index hospitalization.

| **Comorbidity** | **Type** | **ICD-9 Code(s)** | **ICD-10 Code(s)** |
| --- | --- | --- | --- |
| Nephrotic syndrome | Dx | 581.xx | N04.x |
| Congestive heart failure | Dx | 428-428.9x | I50.1, I50.20-I50.23, I50.33, I50.40-I50.43, I50.9 |
| Myocardial infarction | Dx | 410-410.9x, 412-412.9x | I21.x, I22.x, I25.2, I23.x |
| Chronic obstructive pulmonary disease | Dx | 490-496.9x, 500-505.9x, 506.4x | J40, J41.x, J42, J43.9, J44.x, J45.2x, J45.90x, J45.99x, J47.1, J47.9, J60, J61, J62.8, J63.x, J66.x, J64, J67.x, J68.4 |
| Fracture of lower limb | Dx | 820.x | S82.x |
| Inflammatory bowel disease | Dx | 555-556.x | K50-K51.xxx |
| Malignant hypertension | Dx | 401.0, 402-405.xx | I10, I11-I13.xx, I15.x |
| Intubation | Proc | I96.0x-96.5x, 96.6, 96.7x | 09Hxxxx, 099xxxx, 0BHxxxx, 0B9xxxx, 0D9xxxx, 0T7xxxx, 0UHxxxx, 0WHxxxx, 0YQxxxx |

Dx: Diagnostic
Proc, procedural
